# Identifying Cardiac Autoantibody Biomarkers that Predict the Severity of Chagas Cardiomyopathy

**DOI:** 10.1016/j.jacbts.2026.101709

**Published:** 2026-10-09

**Authors:** Heriks Ribeiro, Anton Dmitriev, Antonia Pereira Rosa Neta, Meena Fatah, Diptendu Chatterjee, Lea Campos de Oliveira, Fabiano Castro Albrecht, Hui Tzu Lin Wang, Juliana de Assis S. Gomes, Olgierd Woźniak, Karolina Borowiec, Elżbieta Katarzyna Biernacka, Antonia Claudia Jácome da Câmara, Luciana Sacilotto, Francisco Carlos da Costa Darrieux, Maurício Ibrahim Scanavacca, José Eduardo Krieger, Lúcia Maria da Cunha Galvão, Walderez Dutra, Antonio Luiz Pinho Ribeiro, Edecio Cunha-Neto, Maria Carmo Pereira Nunes, Ester Cerdeira Sabino, Igor Cestari, Andre Ducati Luchessi, Vivian Nogueira Silbiger, Robert M. Hamilton

**Affiliations:** a Department of Clinical Analysis and Toxicology, Federal University of Rio Grande do Norte, Natal, RN, Brazil; b Department of Pediatrics, The Labatt Family Heart Centre and Translational Medicine, The Hospital for Sick Children, Toronto, Ontario, Canada; c Institute of Medical Science, Temerty Faculty of Medicine, University of Toronto, Toronto, Ontario, Canada; d Department of Infectious Disease, Tropical Medicine, University of São Paulo, São Paulo, SP, Brazil; e Dante Pazzanese Institute of Cardiology, São Paulo, SP, Brazil; f Department of Morphology, Institute of Biological Sciences, Federal University of Minas Gerais, Belo Horizonte, MG, Brazil; g Department of Congenital Heart Diseases, Cardinal Stefan Wyszyński National Institute of Cardiology, Warsaw, Poland; h Arrhythmia Unit, Heart Institute (Institute Coração), University of São Paulo Medical School, São Paulo, Brazil; i Laboratory of Genetics and Molecular Cardiology (LGMC), Heart Institute (Institute Coração), University of São Paulo Medical School, São Paulo, Brazil; j Laboratory of Clinical Immunology and Allergy, Heart Institute (InCor), Hospital das Clínicas, Faculdade de Medicina da Universidade de São Paulo, São Paulo, Brazil; k Institute of Investigation in Immunology, National Institute of Science and Technology (iii-INCT), São Paulo, Brazil; l Department of Internal Medicine, School of Medicine, Telehealth Center, Cardiology Service, Hospital das Clínicas, Federal University of Minas Gerais, Belo Horizonte, Minas Gerais, Brazil; m Institute of Parasitology, McGill University, Sainte-Anne-de-Bellevue, Quebec, Canada; n Division of Clinical and Translational Research, Department of Medicine, McGill University, Montreal, Quebec, Canada

**Keywords:** autoantibodies, biomarkers, Chagas cardiomyopathy, Chagas disease, disease severity, ELISA

## Abstract

Chagas cardiomyopathy (ChCM) affects up to 30% of patients with chronic Chagas disease and lacks reliable biomarkers for severity and conduction abnormalities. We profiled cardiac autoantibodies using 2-dimensional gel electrophoresis and mass spectrometry, followed by enzyme-linked immunosorbent assay validation and machine learning analysis. Six autoantibodies linked to glycolysis, adenosine triphosphate metabolism, and sarcomere structure distinguished ChCM stages (area under the curve: 0.87; 95% CI: 0.777-0.977) and a mean classification accuracy (0.84; 95% CI: 0.705-0.935). These findings suggest that an antibody-based panel may enable cost-effective risk stratification and prognostication in ChCM, with prospective validation before clinical application.

## Highlights

• There is a lack of predictive biomarkers for Chagas cardiomyopathy, the most severe manifestation of Chagas disease.
• Six cardiac autoantibodies were associated with Chagas cardiomyopathy severity.
• Cardiac autoantibodies were predictive of abnormalities in cardiac conduction.
• Despite promising findings, validation in larger, diverse study groups is needed to confirm clinical utility.

## Visual Abstract

**Figure:**
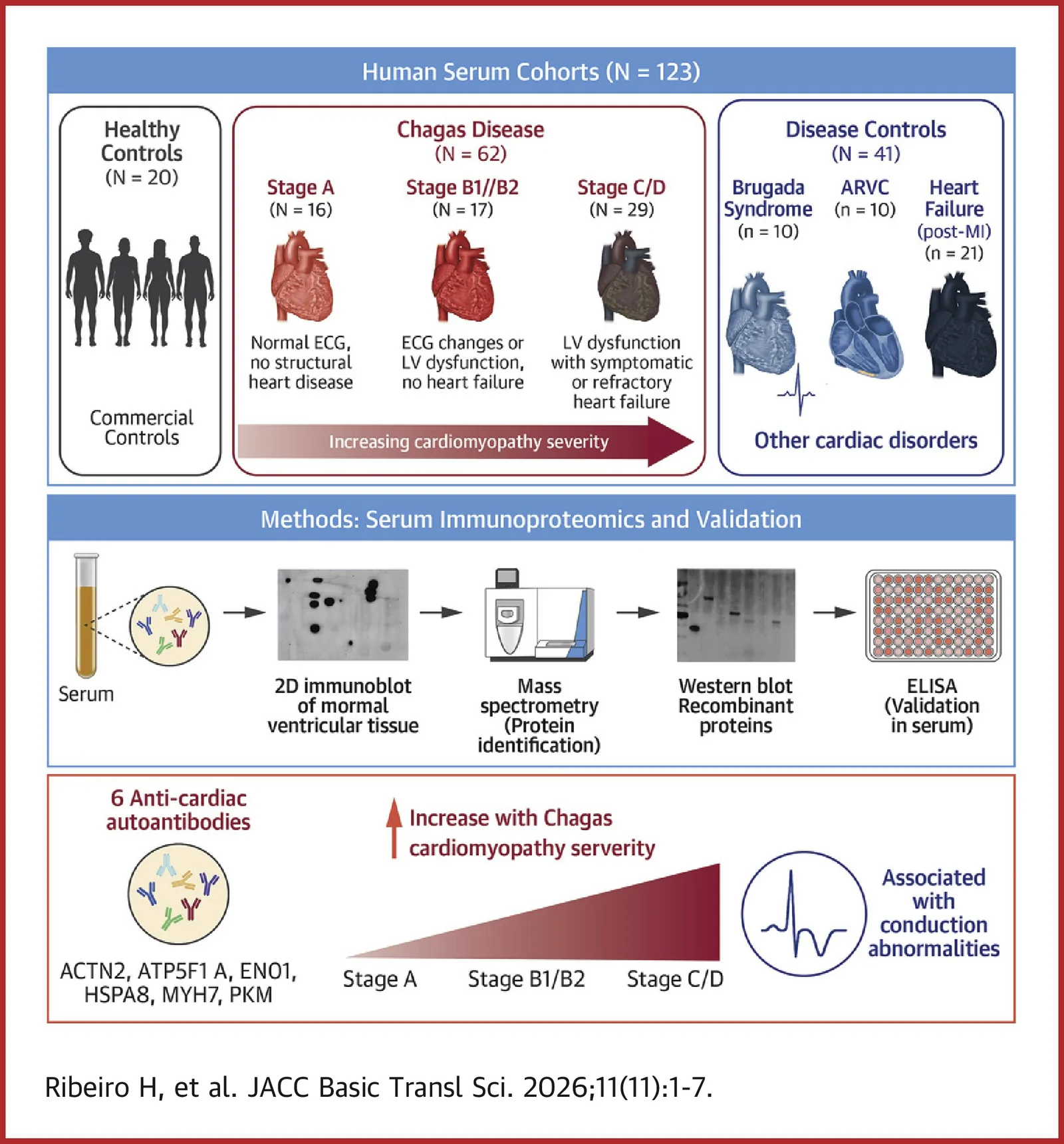

Chagas disease (ChD) is a tropical disease caused by the protozoan parasite *Trypanosoma cruzi*. Endemic to Latin America, it results in more than 8,000 deaths annually.^1^ Recently, human migration and climate-driven changes in vector distribution have spread ChD to an additional 23 countries, including the United States, where endemic, local transmission has been documented.^2^ Among Latin American migrants, seropositivity rates vary widely, reaching up to 53.8% in specialized health care settings in Spain and 2%-4% in North America and Europe.^3^, ^4^, ^5^, ^6^, ^7^

ChD is typically acquired in childhood or early adulthood, most often through vector-borne transmission facilitated by the Triatomine insect, though congenital, transfusion, transplant, and oral transmission also occur.^1^ The disease has an acute and chronic phase, separated by a prolonged latent period. The acute phase, lasting approximately 8 weeks, is typically asymptomatic (95%), with high parasitemia, rare cardiac involvement (1%), and low mortality (<5%).^8^^,^^9^ The indeterminate phase reflects parasite control by innate and Th1-type immune responses, maintaining parasitemia at low or undetectable levels for years (70%).^9^ Over several years to decades, 230%-40% of chronically infected individuals develop Chagas cardiomyopathy (ChCM), the most severe and frequent clinical manifestation of the disease and the main cause of disease-related mortality.^10^

While serological tests that detect anti-*T. cruzi* antibodies are highly reliable for diagnosing ChD (≥95% sensitivity and specificity), predicting which individuals with asymptomatic disease (Stage A, infected but no clinical or subclinical cardiac disease) will progress to ChCM (Stages B-D), and the rate at which this progression will occur, remains a major challenge.^11^, ^12^, ^13^, ^14^, ^15^, ^16^, ^17^, ^18^ This is particularly consequential as ChCM represents a major cause of premature mortality, sudden cardiac arrest, heart failure (HF), and stroke.^11^^,^^12^ Current diagnostic and prognostic methods, including ambulatory electrocardiogram (ECG), echocardiography, cardiac magnetic resonance imaging, and endomyocardial biopsy, primarily detect established structural and functional abnormalities rather than the early molecular and immunological changes associated with ChCM progression.^19^, ^20^, ^21^ Additionally, these modalities are inaccessible in the resource-limited regions where ChD is endemic. Furthermore, currently available cardiovascular biomarkers, such as troponin and brain natriuretic peptide, have limited predictive value in ChCM.^22^ These limitations underscore the need for more sensitive and accessible biomarkers capable of identifying disease progression before irreversible cardiac damage occurs.

Progression from the indeterminate phase to ChCM is driven by a complex interplay between persistent *T. cruzi* infection and host immune responses. *T. cruzi* directly infects cardiomyocytes, where intracellular replication can lead to cell injury and rupture, releasing parasite and host antigens into the surrounding tissue. This process contributes secondarily to chronic inflammation and promotes the activation of immune mechanisms that further amplify myocardial damage.^23^ Persistent infection is associated with both the presence and severity of ChCM, supporting a direct role of the parasite in disease pathogenesis. In parallel, chronic inflammatory responses alter cardiac electrophysiology, disrupt connexin 43 at gap junctions, and induce mitochondrial dysfunction and fibrosis, ultimately compromising conduction and ventricular function, thereby leading to systolic dysfunction.^24^^,^^25^ Additional mechanisms, including T cell–mediated and humoral autoimmunity, are also thought to contribute to disease progression.^11^^,^^12^ Given the growing interest in immune-mediated mechanisms of ChCM, several cardiac autoantibodies have been described in patients with Chagas disease. Functional autoantibodies against β1-/β2-adrenergic and M2-muscarinic receptors have been associated with autonomic dysfunction and arrhythmogenesis.^26^ In addition, classical antibodies against structural and metabolic proteins, including myosin heavy chain 7 (MYH7), α-enolase (ENO1), pyruvate kinase M (PKM), and Heat shock cognate 71 kDa protein (HSPA8), have been reported in different stages of the disease.^27^, ^28^, ^29^, ^30^ Despite these findings, the clinical utility of patient autoantibodies remains uncertain, and their association with disease severity and conduction abnormalities has not been consistently demonstrated. These limitations highlight the need for studies evaluating broader autoantibody signatures and their potential application as biomarkers of ChCM progression.

This study investigated whether anticardiac autoantibody profiles can be used to characterize disease severity in ChCM. Building upon previous studies focused on patient autoantibodies, we employed a proteomics-based discovery and validation workflow using two-dimensional electrophoresis coupled with mass spectrometry (2D-MS) to identify candidate antigens, followed by enzyme-linked immunosorbent assay (ELISA) validation of an independent cohort. We then evaluated the ability of these autoantibodies to discriminate between the different clinical stages of ChCM and cardiac conduction abnormalities. Through this approach, we identified 6 anticardiac autoantibodies that showed differential expression across the different stages of ChCM.

## Methods

### Study design and patient classification

This is a cross-sectional study involved patient recruitment and/or sample analysis across several institutions: the Dante Pazzanese Institute of Cardiology and the Heart Institute (InCor) in Brazil (ethics approval no. 5576264); the National Institute of Cardiology in Poland (institutional ethics committee approval no. 1703); the Hospital Universitário Onofre Lopes – Federal University of Rio Grande do Norte (UFRN) in Brazil (ethics approval no. 65856317.1.0000.5292); and the Hospital for Sick Children (SickKids, ethics approval no. 2973) in Canada. The study protocol adhered to the Declaration of Helsinki and was approved by the Research Ethics Boards of all participating centers. All participants provided written informed consent before enrollment.

A total of 123 individuals were included in this study. Sixty-two patients, aged 18 years or older, of both sexes, with serologically confirmed ChD, were recruited from the Dante Pazzanese Institute of Cardiology and the Heart Institute (InCor), both located in São Paulo, Brazil. Diagnosis was based on clinically validated ELISA. These patients were staged according to the American Heart Association and Brazilian Society of Cardiology guidelines for chronic ChCM, based on a combination of symptoms, electrocardiographic abnormalities, complex ventricular arrhythmias, segmental or global ventricular contractility abnormalities, and left ventricular ejection fraction.^19^ Stage A, referred to as the indeterminate form, includes asymptomatic patients with positive serology for *T. cruzi* and normal ECG and echocardiogram. Stage B represents early cardiomyopathy and is subdivided into B1, characterized by abnormal ECG findings without echocardiographic abnormalities or symptoms, and B2, in which both ECG and echocardiographic abnormalities are present despite the absence of symptoms. Stage C corresponds to the onset of heart failure (HF) symptoms, while Stage D denotes advanced HF with symptoms at rest, often requiring specialized interventions.

For analytical purposes, stages B1 and B2 were grouped as less-advanced cardiomyopathy, whereas stages C and D were grouped as severe cardiomyopathy. In addition, a control group comprising patients with other cardiac disorders was included. Specifically, 10 patients with a confirmed diagnosis of Brugada Syndrome (BrS) were included from the Heart Institute (InCor), Brazil, based on a Shanghai Score >3.5. Ten patients with arrhythmogenic right ventricular cardiomyopathy (ARVC) were recruited from the National Institute of Cardiology in Poland, according to the 2010 revised Task Force Criteria. Twenty-one patients with previous myocardial infarction and left ventricular systolic dysfunction (left ventricular ejection fraction ≤45%), consistent with HF, were recruited from Hospital Universitário Onofre Lopes, Federal University of Rio Grande do Norte (UFRN), Brazil. Additionally, 20 serum samples from healthy volunteers were obtained from Precision for Medicine (HC, health control group). Clinical data for the BrS, ARVC, and HF cohorts have been previously published.^31^^,^^32^ All sera were collected and stored at −80 °C at the sites, then shipped on dry ice to SickKids for further analysis.

### Autoantibody screening by 2D-MS

Candidate anticardiac autoantibodies were identified using the 2D gel electrophoresis protocol previously described by our lab.^33^^,^^34^ Briefly, normal ventricular tissue from material discarded during ventricular septal defect repair was obtained from the Heart Center Biobank at SickKids, then solubilized in CHAPS buffer, sonicated, and subjected to several freeze-thaw cycles. Following this, a 1 μg/μL protein-IEF solution was prepared in ReadyPrep 2-D Starter Kit Sample Rehydration/Sample Buffer (Bio-Rad Laboratories, Inc; Cat#1632106) and applied to ReadyStrip IPG Strips (pH 3-10) (Bio-Rad Laboratories, Inc; Cat#1632000). The proteins were first separated by isoelectric point and subsequently transferred to a 7.5% polyacrylamide gel (Bio-Rad Laboratories, Inc; Cat#456-8021), where they were separated by molecular weight (MW) via standard gel electrophoresis.

Following gel electrophoresis, the proteins were transferred to a nitrocellulose membrane (Bio-Rad Laboratories, Inc; Cat#1620115) and blocked with 3% (w/v) bovine serum albumin (BSA) (MilliporeSigma; Cat#81-053-3) according to the Bio-Rad protocol outlined in their 2D gel electrophoresis procedure. The membranes were then incubated with dilutions of patient sera ranging from 1:100 to 1:5,000. Subsequently, the membranes were developed with goat antihuman HRP-conjugated secondary antibody (Abcam Limited; Cat#98624) at a dilution of 1:50,000 and visualized using the SuperSignal West Dura kit (Thermo Fisher Scientific Inc; Cat#34076) with exposure to autoradiography film (MTC Bio; Cat#A8815).

Using the Western blots, the autoantibody-binding proteins were identified by excising the corresponding spots from a duplicate protein gel run in parallel and performing mass spectrometry at the Molecular Analysis Core Laboratory of the SickKids Proteomics, Analytics, Robotics, and Chemical Biology (SPARC) Biocentre (SickKids Research Institute). Briefly, the proteins were digested into peptides using trypsin overnight. Samples were then analyzed by liquid chromatography-tandem mass spectrometry on an Orbitrap analyzer (Q-Exactive, Thermo Fisher) as previously published. The peptides were identified by database searching against UniProtKB/Swiss-Prot. Raw files acquired from the mass spectrometer were processed using PEAKS software (v.11.0, Bioinformatics Solutions, Inc) and visualized with Scaffold DDA (v.6.5.0, Proteome Software, Inc).

Stratification of proteins with predominantly cardiac expression was performed using The Human Protein Atlas and GeneCards.^35^^,^^36^ Cardiac-related protein associations based on neighboring genes, gene fusions, and coexpression data were identified using STRING. Functional analysis with Gene Ontology was used to determine pathways potentially targeted by antibodies present in ChCM patient sera. Additionally, identified target spots were evaluated based on MW and isoelectric point using the ProtParam database.^37^

### Autoantibody hit validation by western blot

Candidate autoantibody targets identified by 2D-MS were validated by Western blot using recombinant proteins from Creative BioMart. Recombinant antigens were produced in different expression systems depending on the target protein, including *Escherichia coli,* HEK293 cells, wheat germ, yeast, and human-derived sources. Proteins (50 ng each) were resolved on 7.5% polyacrylamide gels and transferred to nitrocellulose membranes (Bio-Rad). Membranes were blocked with 3% BSA (MilliporeSigma) in Tris-buffered saline with polysorbate 20 (Thermo Fisher Scientific), incubated with ChCM patient sera (1:500), and probed with HRP-conjugated secondary antibody (Abcam). Bands were visualized using SuperSignal West Dura (Thermo Fisher Scientific) and autoradiography film (MTC Bio).

### Autoantibody quantification by ELISA

An indirect ELISA was performed following the Abcam protocol.^38^ Briefly, microtiter plates (Thermo Fisher Scientific) were coated with 100 μL of 0.5 ng/μL recombinant protein from Creative BioMart in antigen coating buffer (ImmunoChemistry Technologies) and blocked with 5% BSA (MilliporeSigma). Wells were incubated with human sera (1:5,000) in Neptune Sample Buffer (ImmunoChemistry Technologies) for 2 hours at 25 °C, washed, and probed with goat antihuman HRP-conjugated antibody (ImmunoChemistry Technologies, 1:50,000) for 30 minutes at 25 °C. Plates were developed using 3,3',5,5'-tetramethylbenzidine (TMB) substrate (ImmunoChemistry Technologies), and absorbance at 450 and 600 nm was measured with a Varioskan LUX reader (Thermo Fisher Scientific). Samples were run in duplicate with blanks subtracted for background, and optical density (OD) values were normalized using the Min-Max method.

### Statistical analysis

Continuous data are presented as mean ± SD if parametric and median with 25th-75th percentiles (Q1-Q3) if nonparametric. Categorical data are presented as count (%). Continuous variables (age and ELISA-derived optical density values) were compared across 3 patient groups: indeterminate (Stage A), mild cardiomyopathy (Stages B1/B2), and severe cardiomyopathy (Stages C/D). ELISA values were also compared between HC and BrS, ARVC, HF, and Chagas Stage A patients. For broader analysis, non-ChCM patients included HC, ARVC, BrS, HF, and Stage A, while the ChCM group comprised Stages B1/B2 and C/D. Comparisons between groups were made using the Mann-Whitney *U* test, chi-squared test, 1-way analysis of variance, or the Kruskal-Wallis test followed by the Benjamini-Krieger-Yekutieli post hoc correction as appropriate.

To assess the discriminatory capacity of the selected autoantibody as a predictor of ChCM, comparisons were made between patients at Stage A vs B1/B2, Stage A vs C/D, and individuals with normal ECG vs those with conduction block. A generalized linear model (logistic regression) and receiver operating characteristic (ROC) curve analysis were applied, and the area under the curve (AUC) was calculated with a 95% CI. The optimal cut-off value was determined using Youden's index to maximize the sensitivity + specificity - 1, with AUC values ≥ 0.7 considered indicative of acceptable discriminatory performance. Analyses were conducted using RStudio (v.2024.12.1-563; Posit Software) with the *ggplot2* (v3.5.2) and *pROC* (v.1.18.5) packages, as well as JASP (v.0.19.2). A *P* < 0.05 was considered statistically significant. All plots are presented with Holm-adjusted *P*-values to account for multiple comparisons.

### Development of machine learning methods and evaluation

Supervised machine learning techniques were applied to classify samples as either non-ChCM or ChCM based on proteomic features. All analyses were performed in RStudio (v.2024.12.1-563) using the R packages *caret* (v.7.0-1), *xgboost* (v.1.7.11.1), *randomForest* (v.4.7-1.2), *e1071* (v.1.7-16), and *smotefamily* (v.1.4.0). The data set was randomly split into an 80% training set and a 20% test set for model evaluation. To address class imbalance in the training set, the Synthetic Minority Over-sampling Technique was applied. The numeric features were centered and scaled within the cross-validation folds to prevent data leakage during model training. Four classification algorithms were trained and evaluated: logistic regression (LR), support vector machine with radial kernel (SVM), random forest (RF), and extreme gradient boosting (XGBoost). Hyperparameters were tuned using 5-fold cross-validation, optimizing the area under the ROC curve (AUC). Class probability predictions for non-ChCM and ChCM were extracted for ROC analysis. Model performance was visualized using base R plotting overlays, and feature importance comparisons were presented through segmented plots, enabling interpretability across classifiers.

## Results

### Patient clinical characteristics

A cohort of 62 individuals with ChD was classified into 3 clinical groups based on disease severity: Stage A (n = 16), Stage B1/B2 (n = 17), and Stage C/D (n = 29). Clinical characteristics are summarized in Table 1. The overall mean aged was 61.9 ± 11.1 years, with significant differences between groups (*P* = 0.014), and 54.8% of participants were women. Regarding electrocardiographic findings, right bundle branch block (RBBB) was present in 37.5% and 43.7% of patients in the B1/B2 and C/D groups, respectively, while left bundle branch block (LBBB) was observed in 25.0% of patients in both groups. Atrial fibrillation was noted in 1 patient in stage C/D, and second-degree atrioventricular block (AVB-2) occurred in 1 patient in B1/B2 and in 2 patients in C/D. Functional capacity, assessed by NYHA classification, showed progressive worsening with advancing disease stages (*P* = 0.030). In the B1/B2 group, NYHA functional classes I, II, and III were observed in 50.0%, 33.3%, and 16.7% of patients, respectively, while in the C/D group, 24.1% were in the most advanced class. Left ventricular ejection fraction differed significantly across stages (*P* = 0.001), with mean values of 67.0 ± 3.8 in group A, 57.2 ± 10.1 in B1/B2, and 28.7 ± 6.2 in C/D. Consistent with this, 11.8% of patients in B1/B2 had a pacemaker or implantable cardioverter-defibrillator, compared to 24.1% with pacemakers and 24.1% with implantable cardioverter-defibrillators in the severe group. Clinical data for the control groups with other disorders were added to the clinical data table (Supplemental Tables 2 to 4).

**Table 1:** Clinical Characteristics of Chagas Disease Patients Stratified According to ChCM Clinical Stages

Cohort (n)	All (62)	A (16)	B1/B2 (17)	C/D (29)	*P*
Age, y	61.9 ± 11.1 61.0 [55.3-70.0]	60.9 ± 7.3 60.0 [57.8-65.5]	67.8 ± 11.9 70.0 [64.0-76.0]	59.0 ± 11.3 58.0 [53.0-66.0]	**0.014**^a^
Female % (n)	54.8% (34)	75.0% (12)	47.0% (8)	48.3% (14)	0.17^b^
ECG					**0.001**^b^
AF, % (n)	2.1% (1)	0% (0)	0% (0)	6.2% (1)	
AVB-2, % (n)	6.2% (3)	0% (0)	6.2% (1)	12.5% (2)	
LBBB, % (n)	16.6% (8)	0% (0)	25% (4)	25% (4)	
RBBB, % (n)	27.1% (13)	0% (0)	37.5% (6)	43.7% (7)	
NS-PAT	8.3% (4)	0% (0)	25% (4)	0% (0)	
NSVT	4.1% (2)	0% (0)	6.2% (1)	6.2% (1)	
SVT	2.1% (1)	0% (0)	0% (0)	6.2% (1)	
Normal ECG, % (n)	33.3% (16)	100% (16)	0% (0)	0% (0)	
NYHA Class					**0.030**^b^
I, % (n)	30.4% (14)	80.0% (4)	50.0% (5)	13.8% (4)	
II, % (n)	39.1% (18)	20.0% (1)	33.3% (4)	44.8% (13)	
III, % (n)	15.2% (7)	0% (0)	16.7% (2)	17.2% (5)	
IV, % (n)	15.2% (7)	0% (0)	0% (0)	24.1% (7)	
Cardiac device					**0.003**^b^
Pacemaker, % (n)	12.9% (8)	0% (0)	5.9% (1)	24.1% (7)	
ICD % (n)	12.9% (8)	0% (0)	5.9% (1)	24.1% (7)	
No use, % (n)	74.2% (46)	100% (16)	88.2% (15)	51.7% (15)	
LVEF	46.4 ± 18.44 46.0 [29.0-64.0]	67.0 ± 3.81 67.0 [64.0-68.3]	57.2 ± 10.1 59.0 [48.0-65.0]	28.7 ± 6.25 28.0 [25.0-34.0]	**0.001**^a^
SAH, % (n)	61.9% (34)	62.5% (10)	70.6% (12)	54.5% (12)	0.56^b^
DM, % (n)	27.3% (15)	31.2% (5)	23.5% (4)	27.3% (6)	0.92^b^
FH of ChD, % (n)	45.4% (25)	50.0% (8)	64.7% (11)	27.3% (6)	0.057^b^

Subject numbers are shown in parentheses. Complete data were available for stage, sex, age, NYHA functional class, cardiac device, and LVEF (62/62, 100%); SAH, DM, and FH of ChD (55/62, 88.7%); and ECG (54/62, 87.1%). Continuous variables are presented as mean ± SD and median [Q1-Q3]; categorical variables as percentage (count). **Bold** indicates statistical significance (*P* < 0.05).

AF = atrial fibrillation; AVB-2 = atrioventricular block type 2; ChD = Chagas disease; DM = diabetes mellitus; ECG = electrocardiogram; FH = family history; ICD = implantable cardioverter defibrillator; LBBB = left bundle branch block; LVEF = left ventricular ejection fraction; NS-PAT = non-sustained atrial tachycardia; NSVT = non-sustained ventricular tachycardia; RBBB = right bundle branch block; SAH = systemic arterial hypertension; SVT = sustained ventricular tachycardia.

a Kruskal-Wallis’ test.

b Fisher's exact test via Monte Carlo simulation.

### Discovery platform by 2D gel electrophoresis and western blot validation

We analyzed 4 serum samples by 2D gel electrophoresis to detect anticardiac antibodies: 3 from patients with advanced ChCM (Stage C/D) and 1 from a patient with moderate disease (Stage B1/B2) (Figures 1A to 1D). This analysis revealed 10 consistent protein spots in the same locations in all 4 ChCM samples, which were subsequently subjected to liquid chromatography-tandem mass spectrometry for protein identification. Following quality control (1% false discovery rate [FDR], ≥2 unique peptides, >20% sequence coverage), the data were filtered to retain high-confidence cardiac proteins, compiled from UniProt, neXtProt, and the Human Protein Atlas. The selected proteins were further analyzed using bioinformatics resources, including STRING and Gene Ontology, to confirm cardiac-specific expression and identify potential associations with cardiac-related pathways, protein–protein interactions, and disease mechanisms. This ensured their relevance to heart function and the pathology of *T. cruzi* infection. From this process, 8 key proteins were identified: α-actinin-2 (ACTN2), ACTG1, adenosine triphosphate synthase F1 subunit α (ATP-5F1A), ENO1, HSPA8, MYH7, PKM, and tubulin beta chain (TUBB) (protein–protein interaction enrichment *P* = 7.0 × 10^−4^; Figure 1G). Tissue expression in the cardiovascular system was significant (FDR = 4.3 × 10^−5^). These proteins are associated with essential biological processes, including ATP metabolism (GO:0046034, FDR = 1.2 × 10^−3^) and sarcomere organization (GO:0045214, FDR = 1.2 × 10^−3^), with subcellular localization in the actin filament (GO:0005884, FDR = 0.049) (Figure 1F).

**Figure 1:**
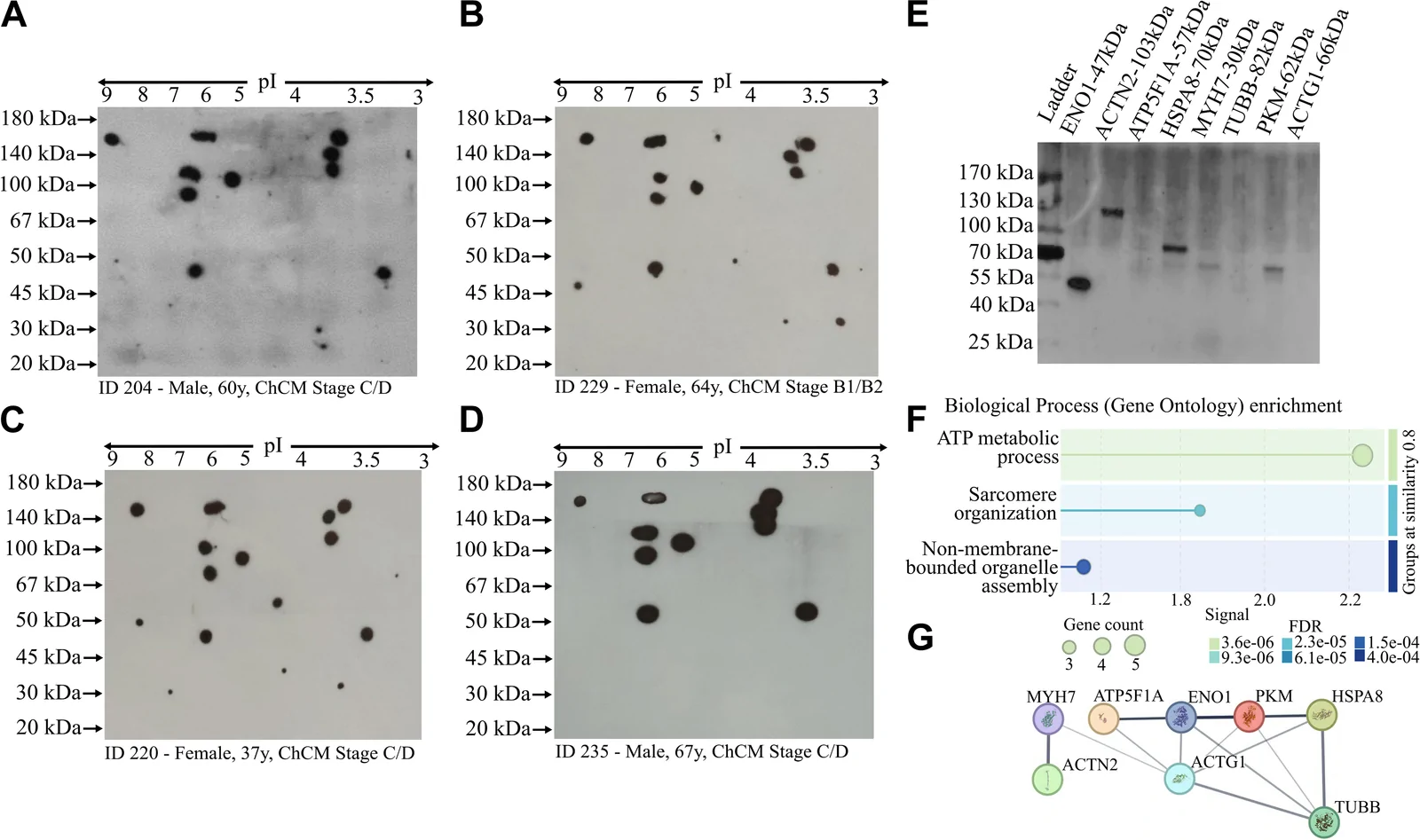
Autoantibody Reactivity and Protein Networks in Chagas Cardiomyopathy (A to D) Two-dimensional electrophoresis showing immunoglobulin G reactivity profiles in sera from 4 patients with chronic Chagas cardiomyopathy (ChCM) at stages B1/B2 and C/D. Proteins from healthy human cardiac tissue were separated by isoelectric point and sodium dodecyl sulfate-polyacrylamide gel electrophoresis (SDS-PAGE), and membranes were incubated with patient sera to identify cardiac targets of circulating autoantibodies. A similar binding pattern with 10 spots was observed, suggesting common reactivity among patients. (E) Western blot analysis of anticardiac responses in Chagas disease. (F) Functional enrichment analysis of the protein–protein interaction network generated with Search Tool for the Retrieval of Interaction Genes/Proteins (STRING) 12.0, where bubble size reflects the number of proteins associated with each pathway or Gene Ontology term. (G) Protein–protein interaction network of 8 proteins, generated with STRING 12.0 (confidence score 0.700), with nodes representing proteins and edges representing predicted interactions; node colors indicate protein functions. ACTG1 = actin gamma 1; ACTN2 = α-actinin-2; ATP5F1A = adenosine triphosphate synthase F1 subunit α; ENO1 = α-enolase; HSPA8 = heat shock cognate 71 kDa protein; MYH7 = myosin heavy chain 7; PKM = pyruvate kinase M; TUBB = tubulin beta chain.

The proteins were purchased from Creative BioMart in the following commercial forms: ACTG1-216H, ACTG1-6898H, ACTN2-1023H, ATP5FA1-2238H, ENO1-1814H, HSPA8-069H, HSPA8-4712H, MYH7-8020H, MYH7-8021H, PKM-009H, and TUBB-3923H, totaling 11 targets for Western blot validation. Detailed information about the recombinant proteins, including MW, degree of cardiac expression, and source, is provided in Table 2. As an initial validation step, Western blotting was performed to evaluate the immunoreactivity of all 11 recombinant proteins identified by 2D-MS. Six proteins, ACTN2-1023H, ATP5F1A-2238H, ENO1-1814H, MYH7-8021H, HSPA8-069H, and PKM-009H, had clear and specific immunoreactivity and were therefore selected for subsequent ELISA analyses. The remaining proteins did not exhibit detectable or sufficiently specific reactivity under the experimental conditions and were discarded as false positives and not evaluated further (Figure 1E).

**Table 2:** List of Autoantibody-Targeted Cardiac Proteins: Properties and Tissue Expression in Chagas Cardiomyopathy

Protein	Symbol	Cat Number	UniProt ID	pI	MW	Cell compartment^b^	Heart Expression^c^	Expression System
Actin gamma 1	ACTG1	216H	P63261	5.6	66.99 kDa	Cytoplasm, Nucleus	Low	Wheat Germ
Actin gamma 1	ACTG1	6898H	P63261	5.6	43 kDa	Cytoplasm, Nucleus	Low	*E. coli*
Actinin alpha 2	ACTN2	1023H	P35609	5.31^a^	103.7 kDa	Cytoplasm, Z-lines, Nucleus, Cell Membrane	High	HEK 293
ATP Synthase F1 Subunit Alpha	ATP5F1A	2238H	P25705	8.28^a^	57.2 kDa	Mitochondria, Cell Membrane	High	Yeast
Alpha-enolase	ENO1	1814H	P06733	7.01^a^	47.1 kDa	Cell membrane, Cytoplasm, M-lines, Nucleus	Low	*E. coli*
Tubulin beta chain	TUBB	3923H	P07437	4.78^a^	32.2 kDa	Cytoplasm, Nucleus	Low	*E. coli*
Heat shock cognate 71 kDa protein	HSPA8	4712H	P11142	5.68^a^	73.1 kDa	Cytoplasm, Nucleus, Cell Membrane	High	*E.coli*
Heat shock cognate 71 kDa protein	HSPA8	069H	P11142	5.68^a^	72.9 kDa	Cytoplasm, Nucleus, Cell Membrane	High	*E. coli*
Myosin heavy chain 7	MYH7	8020H	P12883	5.3	30.5 kDa	Cytoplasm, Nucleus, Mitochondria	High	*E. coli*
Myosin heavy chain 7	MYH7	8021H	P12883	5.63^a^	22 kDa	Cytoplasm, Nucleus, Mitochondria	High	*E. coli*
Pyruvate kinase PKM	PKM	009H	P14618	8.22^a^	60.1 kDa	Cytoplasm, Nucleus, Cell Membrane	Medium	*E. coli*

Isoelectric point estimated using the ExPASy ProtParam tool.

kDa = kilodalton; MW = molecular weight; pI = isoelectric point.

a Data without were provided by Creative Biomart.

b Data from GeneCards.

c Data from The Human Protein Atlas (access: July 25, 2026). ACTG1-6898H and ACTG1-216H share the same length (1-375 aa) but differ in source, molecular weight (43 vs 66.99 kDa), and tag (His vs GST). HSPA8-069H and HSPA8-4712H are both *E. coli*–expressed and His-tagged, with slight differences in length (2-646 vs 1-646 aa) and mass (72.9 vs 73.1 kDa). MYH7-8021H (1777-1935 aa, 22 kDa) and MYH7-8020H (1268-1516 aa, 30.5 kDa) also differ in range and molecular weight.

### Anticardiac antibodies ELISA

Following the optimization of ELISA assays targeting 6 proteins, statistically significant differences in OD values were observed between HC and patients with ChD. Among the proteins analyzed, ENO1 stood out, exhibiting distinct OD profiles also between other patient groups: ARVC (*P* = 1.0 × 10^−4^) and HF (*P* = 6.0 × 10^−4^), underscoring its potential relevance across cardiomyopathic conditions (Figures 2A to 2F). To further explore protein expression across disease ChD stages. When contrasting indeterminate-stage patients (A) with those in the moderate stage (B1/B2), significant differences were observed for ENO1-1814H (*P* = 0.0073). More pronounced distinctions emerged between indeterminate (A) and severe (C/D) stages, where ACTN2-1023H, ATP5F1A-2238H, ENO1-1814H, MYH7-8021H, and PKM-009H all showed significance (*P*-values ranging from 0.0016 to 0.0096). No significant differences were found between moderate (B1/B2) and severe (C/D) stages, except for ACTN2 (*P* = 0.023). Furthermore, all proteins significantly discriminated indeterminate-stage (A) patients from the control group (Figures 3A to 3F). Additionally, when comparing the severe-stage group (C/D) with patients diagnosed with BrS, ARVC, and HF, significant differences were observed for all comparisons across all target proteins (Supplemental Figures 1A to 1F).

**Figure 2:**
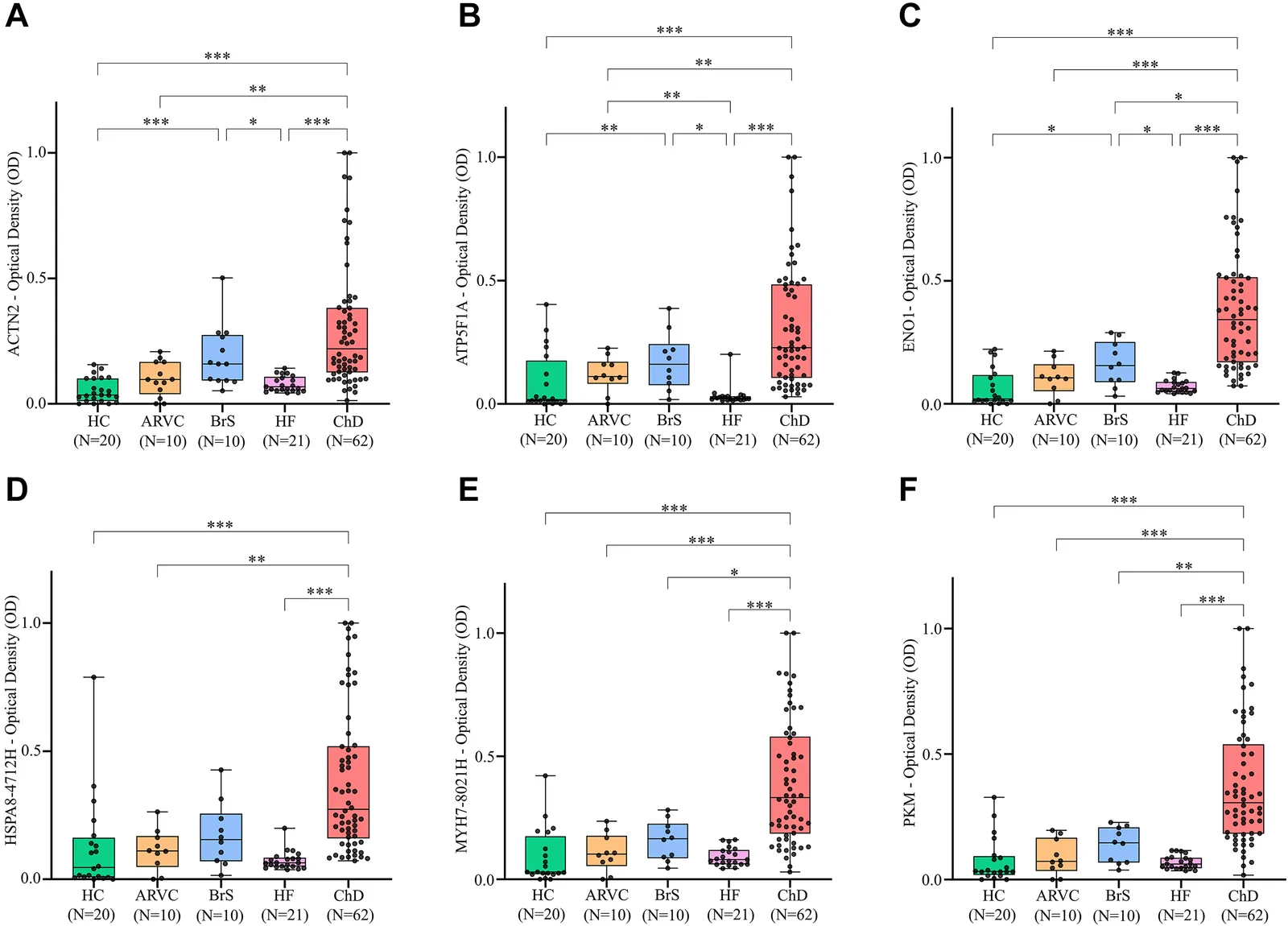
Anticardiac Antibodies Across Healthy and Cardiomyopathy Cohorts (A to F) Box plots display individual data points, medians, interquartile ranges, and min–max whiskers for anticardiac antibodies against α-actinin-2, α-enolase, heat shock cognate 71 kDa protein-4712H, myosin heavy chain 7-8021H, and pyruvate kinase M across healthy controls (HC), arrhythmogenic right ventricular cardiomyopathy (ARVC), Brugada syndrome (BrS), ischemic heart failure (HF), and Chagas disease (ChD). Statistical analysis used the Kruskal-Wallis test with post hoc pairwise comparisons via the Benjamini, Krieger, and Yekutieli method. Adjusted *P*-values and sample sizes (N) are shown. ∗*P* < 0.05; ∗∗*P* < 0.01; ∗∗∗*P* < 0.001.

**Figure 3:**
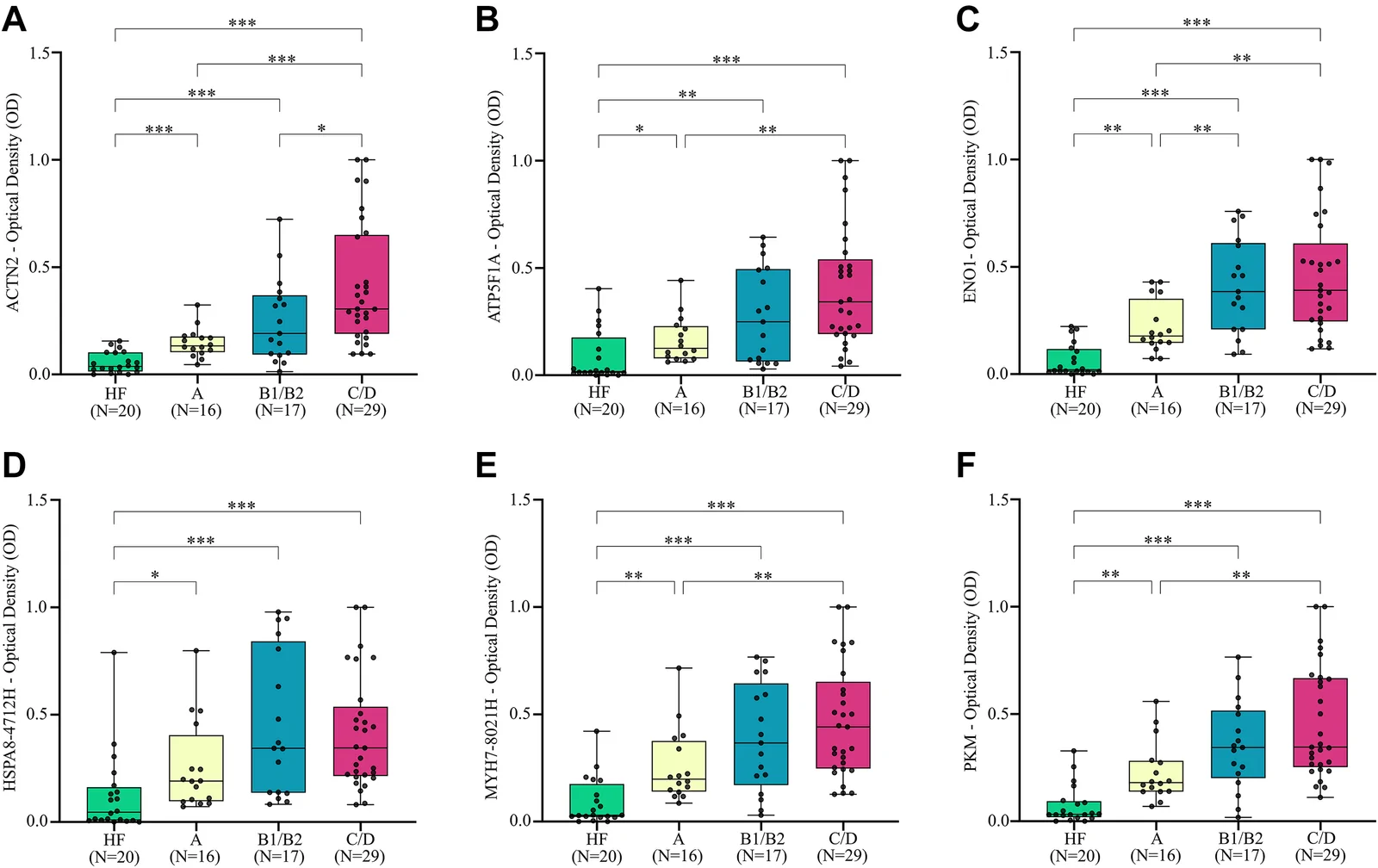
Anticardiac Antibodies Across Healthy Controls and Chagas Stages (A to F) Box plots display individual data points, medians, interquartile ranges, and min–max whiskers for anticardiac antibodies against α-actinin-2, adenosine triphosphate synthase F1 subunit α, α-enolase, heat shock cognate 71 kDa protein-4712H, myosin heavy chain 7-8021H, and pyruvate kinase M in healthy controls (HC) and Chagas disease (ChD), stratified by Chagas cardiomyopathy (ChCM) stages (A, B1/B2, C/D). Statistical analysis used the Kruskal-Wallis test with post hoc pairwise comparisons via the Benjamini, Krieger, and Yekutieli method. Adjusted *P*-values and sample sizes (N) are shown. ∗*P* < 0.05; ∗∗*P* < 0.01; ∗∗∗*P* < 0.001.

### Anticardiac antibody panels across Chagas disease stages

Generalized linear models were applied to identify optimal cutoff points distinguishing clinical stages based on protein expression levels. Odds ratios were calculated for every 0.1 unit increase in OD. ENO1-1814H exhibited statistically significant discriminatory power between ChD stage A and stages B1/B2 (OR = 1.88; 95% CI: 1.14-3.11) (Figure 4A). Detailed cutoff values derived from the Youden index are provided in the Supplementary Material (Supplemental Table 1). The ROC curve analysis further supported these findings. The highest AUC was observed for the combined protein panel (AUC: 0.79; 95% CI: 0.632-0.963), followed by ENO1 (AUC: 0.76; 95% CI: 0.600-0.936) and PKM (AUC: 0.68; 95% CI: 0.488-0.876) (Figure 4D). All proteins except HSPA8-4712H discriminated stage A vs C/D, with ACTN2-1023H showing the strongest effect (OR = 3.1; 95% CI: 1.38-7.15) (Figure 4B and 4E). A similar pattern was observed when evaluating patients who exhibit conduction abnormalities, such as AVB-2, RBBB, and LBBB. The ACTN2-1023H displayed the highest OR (OR = 2; 95% CI: 1.13-3.53), followed by ENO1 (OR = 1.96; 95% CI: 1.22-3.17) (Figure 4C). The combined 6-protein panel achieved an overall AUC of 0.84 (95% CI: 0.724-0.964). When analyzed individually, ENO1 and PKM reversed in performance (AUC: 0.79; 95% CI: 0.651-0.932) and (AUC: 0.76; 95% CI: 0.615-0.919), respectively (Figure 4F).

**Figure 4:**
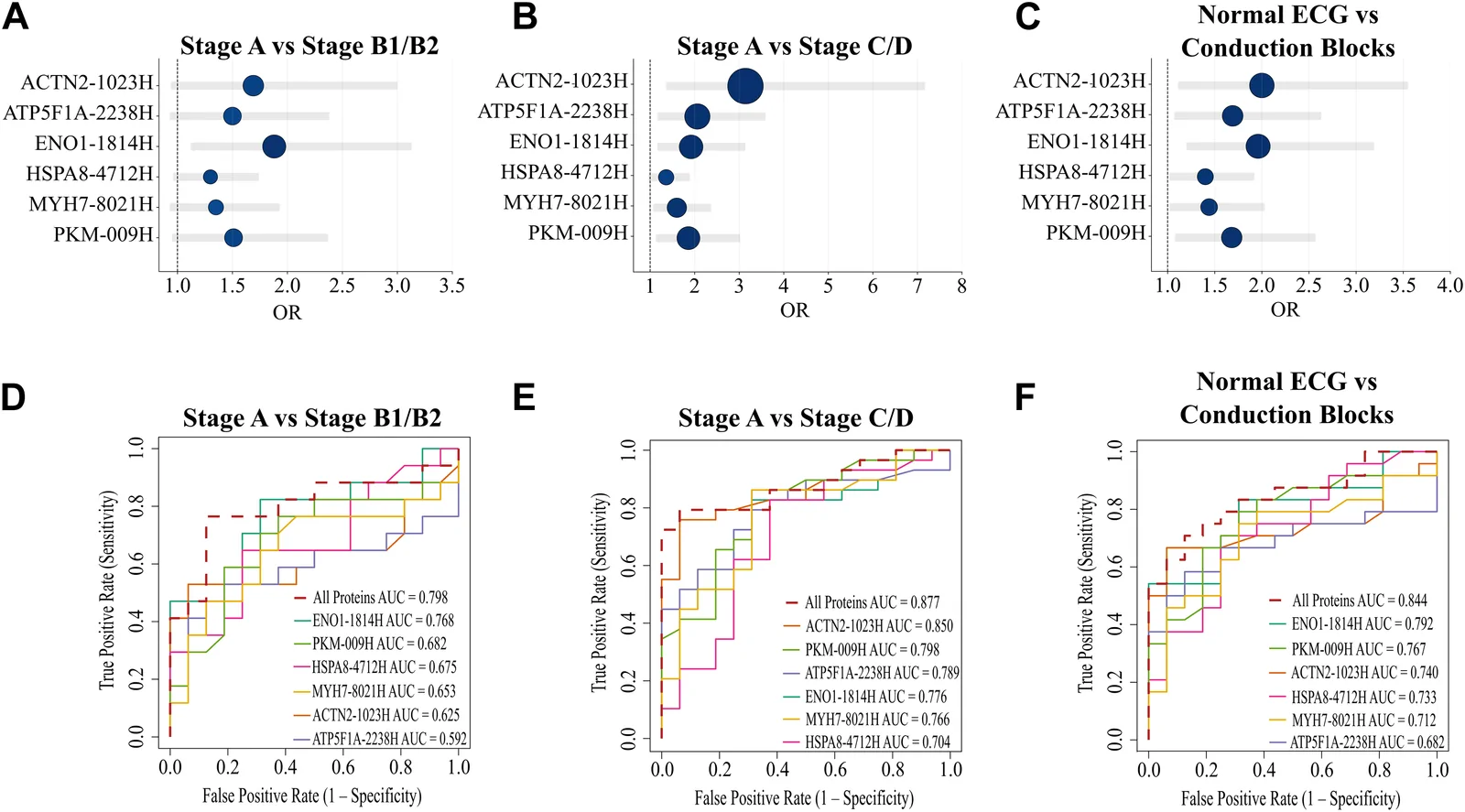
Predictive Performance of Autoantibodies Across Chagas Stages and Conduction Blocks (A to C) Forest plots of logistic regression models for candidate autoantibody markers across Chagas disease (ChD) stages, showing odds ratios (blue circles) and 95% CI (gray bars) per 0.1 optical density increment. Youden cut-offs were used to optimize sensitivity and specificity between stages. (D to F) Receiver operating characteristic curves illustrating model performance for distinguishing ChD stages A, B1/B2, and C/D, with area under the curve (AUC) values indicating overall discrimination. ACTN2 = α-actinin-2; ATP = adenosine triphosphate synthase F1 subunit α; ATP5F1A = adenosine triphosphate synthase F1 subunit α; ECG = electrocardiogram; ENO1 = α-enolase; HSPA8 = heat shock cognate 71 kDa protein; MYH7 = myosin heavy chain 7; PKM = pyruvate kinase M.

### Machine learning for prediction of Chagas cardiomyopathy

To evaluate the discriminatory power of candidate biomarkers, ELISA assays were conducted for 6 targeted proteins using samples classified as either non-ChCM or ChCM. All 6 proteins exhibited statistically significant differences between the 2 groups (*P* < 0.001), highlighting their diagnostic potential (Figures 5A to 5F). Additionally, 4 machine learning algorithms (LR, SVM, RF, and XGBoost) were applied to evaluate the ability of these biomarkers to distinguish between non-ChCM and ChCM cases. The use of multiple computational approaches enabled validation of classification performance and identification of biomarkers that consistently contributed to discrimination across models, providing evidence that the observed discriminatory signal was not dependent on a specific algorithm (Figure 6). All 4 models showed comparable discriminative performance. SVM and RF achieved the highest values (AUC = 0.89), while LR and XGBoost both reached an AUC of 0.87. Accuracy was 79% for SVM, RF, and LR (95% CI: 0.578-0.929) and 70% for XGBoost (95% CI: 0.489-0.874) (Figure 6A). Variable importance differed across models: in the SVM model, PKM, followed by ENO1, contributed most strongly to classification, whereas in the LR model ACTN2 was the leading contributor (Figure 6B). Overall, the consistent classification performance across models, together with the recurrent identification of ACTN2, PKM, and ENO1 among the strongest contributors, supports the biological relevance of these biomarkers for distinguishing ChCM and non-ChCM cases.

**Figure 5:**
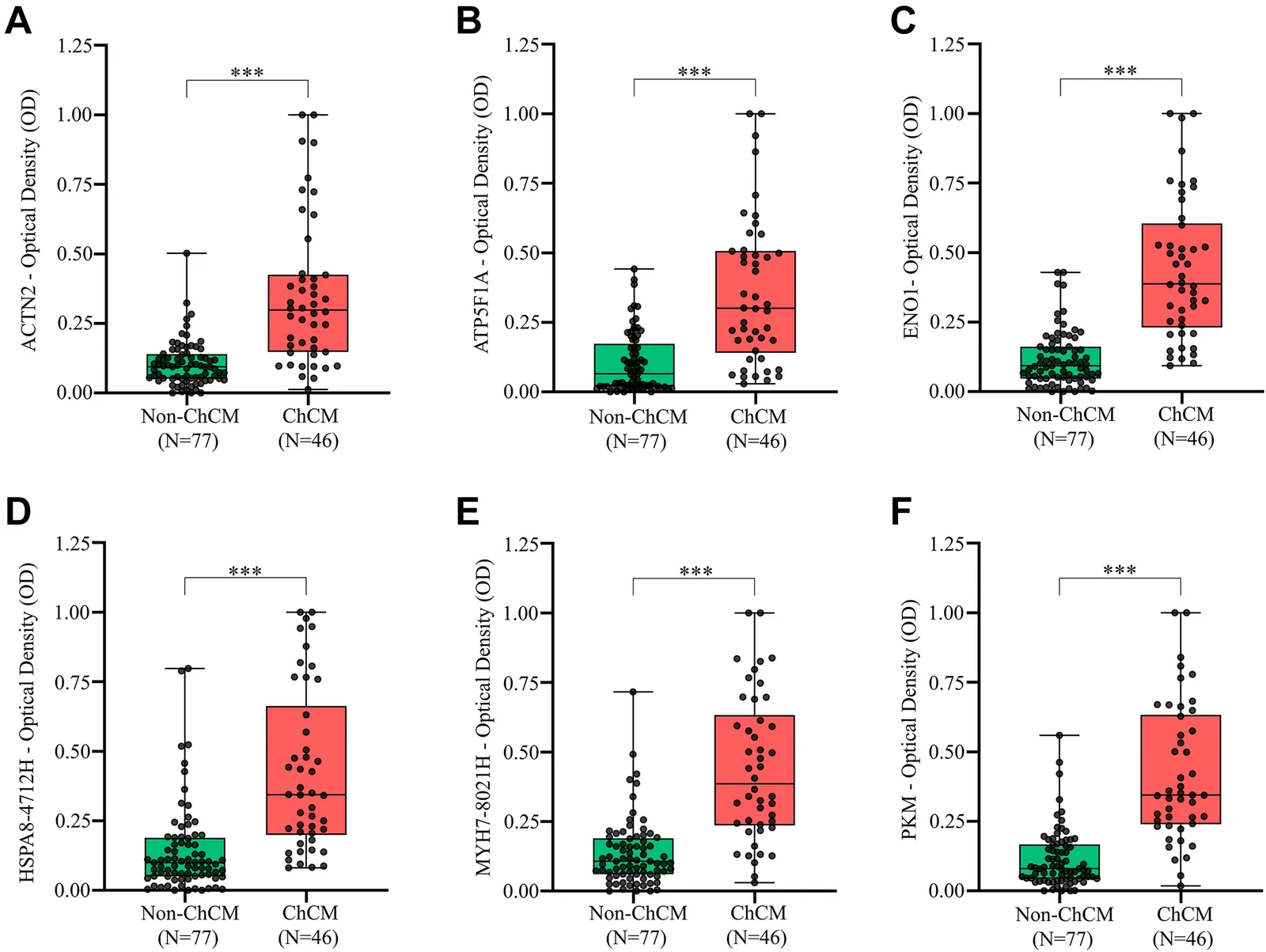
Anticardiac Antibody Levels in Non-Chagas Cardiomyopathy (ChCM) and ChCM Groups (A to F) Box plots display individual data points, medians, interquartile ranges, and min–max whiskers for anticardiac antibodies against α-actinin-2 (ACTN2), adenosine triphosphate synthase F1 subunit α (ATP5F1A), heat shock cognate 71 kDa protein (HSPA8)-4712H, α-enolase (ENO1), myosin heavy chain 7 (MYH7)-8021H, and pyruvate kinase M (PKM) in non-ChCM and ChCM groups. Statistical analysis used the Kruskal-Wallis test with post hoc pairwise comparisons via the Benjamini, Krieger, and Yekutieli method. Adjusted *P*-values and sample sizes (N) are indicated. The non-ChCM group includes healthy controls, arrhythmogenic right ventricular cardiomyopathy, Brugada syndrome, ischemic heart failure, and Chagas disease (ChD) Stage A, while the ChCM group includes ChD stages B1/B2 and C/D. ∗*P* < 0.05; ∗∗*P* < 0.01; ∗∗∗*P* < 0.001.

**Figure 6:**
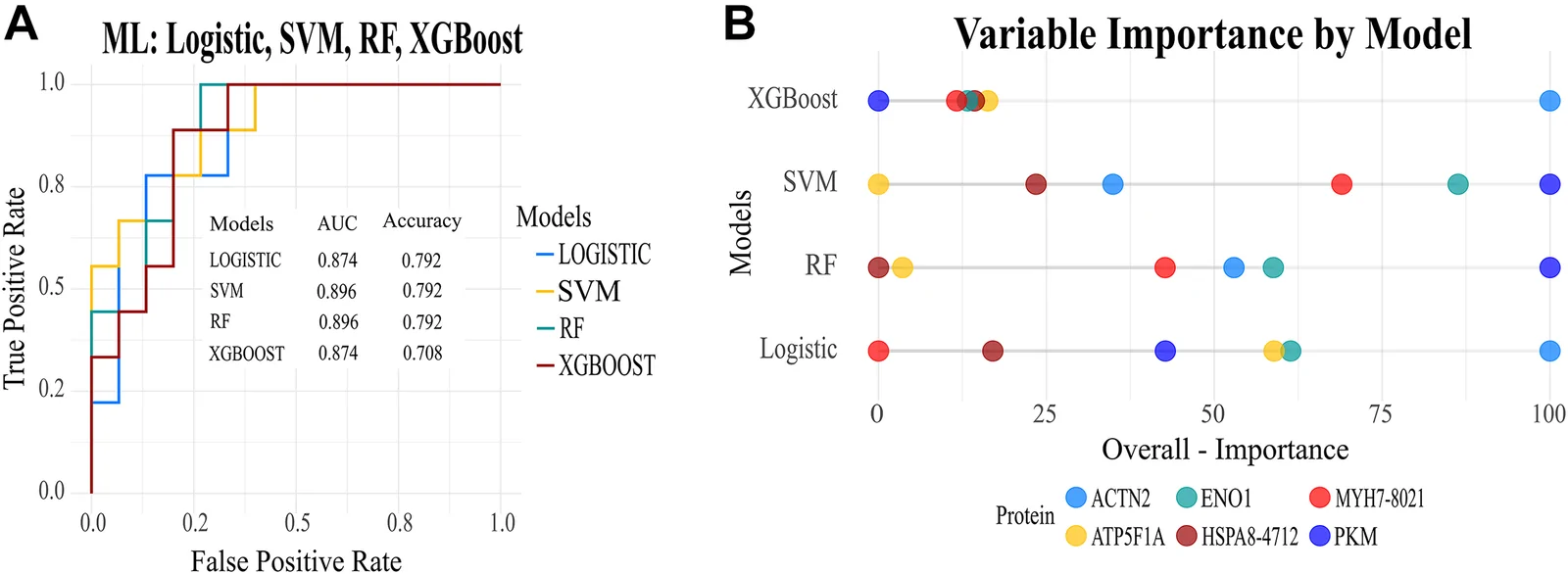
Machine Learning Models for Identifying Chagas Cardiomyopathy (A) Receiver operating characteristic curves comparing 4 machine learning models, logistic regression (LR), random forest (RF), support vector machine (SVM), and XGBoost, for distinguishing non-Chagas cardiomyopathy (non-ChCM) from ChCM, with area under the curve and accuracy reported. (B) Overall variable importance of protein markers (α-actinin-2 [ACTN2], adenosine triphosphate synthase F1 subunit α [ATP5F1A], heat shock cognate 71 kDa protein [HSPA8]-4712H, α-enolase [ENO1], myosin heavy chain 7 [MYH7]-8021H, and pyruvate kinase M [PKM]) across models, reflecting each protein's contribution to classification performance.

## Discussion

ChD is a major cause of infectious cardiomyopathy in Latin America, accounting for substantial morbidity and mortality, and is increasingly recognized as a global health concern. A variety of mechanisms, including humoral autoimmunity, are hypothesized to cause cardiomyopathy with HF and arrhythmia in chronic ChD. Using serum samples derived from patients with ChD, we identified a novel panel of 6 anticardiac autoantibodies, ENO1, PKM, ACTN2, MYH7, ATP5F1A, and HSPA8, whose relative levels increase with worsening ChCM and combined provide strong predictive power in differentiating stage A patients from those in stage B1/B2 and C/D, as well as those with and without conduction abnormalities. This unique antibody profile may serve as a new biomarker-driven approach to track disease severity and manage ChCM manifestations in patients with ChD.

In this study, we demonstrated that ENO1, a glycolytic enzyme that translocates to the plasma membrane in the presence of an inflammatory stimulus, was the best autoantibody for discriminating between clinical stages A and B1/B2.^39^ Furthermore, it accurately identified individuals with conduction abnormalities, underscoring its potential as one of the strongest prognostic biomarkers for ChCM. This is in line with previous research that identified ENO1 autoantibodies in patients with ChD across all clinical stages, with worsening clinical stage broadly correlating with a relative increase in autoantibody levels, as measured by binding to oxidized ENO1 in *T. cruzi* infection.^29^ With other research highlighting that patient sera recognizes recombinant *T. cruzi enolase* with 55% sequence homology to human β-enolase (ENO3) and that antitroponin I IgG (cTnIAAb) binds ENO1 exposed on the cardiomyocyte membrane leading to PI3K-Akt-mediated apoptosis, collagen deposition, and adverse ventricular remodeling, this finding represents another clue to the mechanism of disease causing ChCM.^40^

We also identified a second autoantibody target involved in glycolysis, PKM, which closely paralleled ENO1; its predictive power was nearly equal to that of ENO1 for discriminating clinical stages A and B1/B2 and normal ECG from those with conduction blocks. Known to play a fundamental role in cardiac function, dysregulation of the PKM protein has been implicated in HF, with the circulating form increased in patients with a poorer prognosis and an increase in the tetrameric form improving mitochondrial function and reducing the progression of right ventricular failure.^41^, ^42^, ^43^ As with ENO1, PKM autoantibodies were also detected in patients with ChD across all clinical stages, with a more severe prognosis broadly correlating with a relatively higher anti-PKM level as measured by binding in an oxidizing environment similar to that induced by *T. cruzi* infection.^29^ Furthermore, patients with ChD with digestive disease had the highest anti-PKM2 IgG titers among healthy, indeterminate, ChCM, and ChCM-digestive disease individuals, implying that these autoantibodies may be associated with the development of digestive tract lesions. Similarly, anti-PKM titers increased with cardiac stage in our cohort, paralleling disease severity.^30^ The prevailing notion is that the gastrointestinal disorders of ChD arise due to destruction of the autonomic neurons, which, combined with the elevated titers, can lead one to surmise that PKM contributes to ChCM etiology in a similar manner.^44^

It is worth noting that while both PKM and ENO1 have been associated with glycolytic exhaustion during the progression of ChCM, the mechanisms by which autoantibodies to PKM are formed have not been characterized, and unlike with ENO1, no *T. cruzi* protein with significant sequential, structural, or functional homology has been identified.^44^ Thus, even though an undiscovered PKM homolog in *T. cruzi* remains a possibility given that glycolysis and its enzymes are generally well conserved across the animal kingdom, it is equally reasonable to hypothesize that PKM is an immune-privileged autoantigen or cryptic epitope exposed upon acute myocytolysis by *T. cruzi* acidic hemolysins (TC-TOX and LTY-1), and it is this that leads to the formation of anti-PKM autoantibodies.^8^^,^^45^ Then, subsequent sporadic recirculation of the parasite perpetuates inflammation and tissue damage, potentially helping to further release the immunogenic autoantigen and maintain autoantibody levels in the absence of localized parasitemia.^8^

One of 2 cytoskeletal elements we identified, ACTN2, which is the principal crosslinking protein of the Z-disc connecting F-actin to titin to modulate force transmission and sarcomeric alignment in striated muscle fibers, was the strongest discriminator of advanced disease, best distinguishing stage A from stages C/D. While the ACTN2 protein and its link to congenital cardiomyopathy have been extensively studied, there are no reports of an association between ACTN2 and autoimmunity in the cardiac context to the best of our knowledge. A possible pathogenic variant in ACTN2 has been associated with severe ventricular fibrillation and a phenotype resembling hypertrophic cardiomyopathy with diastolic dysfunction, providing a potential explanatory link to its role as a biomarker of both arrhythmia and advanced cardiomyopathy.^46^

MYH7 autoantibodies showed only moderate predictive performance for both clinical severity and conduction abnormalities. Nonetheless, the increase in anti-MYH7 with worsening ChCM that we observed is in line with other related research that reported increasing levels of anti-B13 antibodies, a cross-reactive antibody that binds both the cardiac myosin heavy chain and the *T. cruzi* antigen B13, with worsening ChCM, although they obtained an AUC = 0.87.^27^^,^^28^ Another work further demonstrated that there was a significant difference in anti-B13 antibodies between indeterminate stage patients and those with stage C ChCM and noted that it shows promise as a biomarker for identifying individuals who are in the clinically asymptomatic stage and are at-risk of developing severe ChCM.^47^ While the individual AUC in their study was only 69%, comparable to the moderate values we obtained, that improved to 83% in combination with anti-β1-adrenoreceptor autoantibodies, further supporting our methodological approach of combining several autoantibody biomarkers together to generate a prognostic panel that is highly specific to ChCM. By combining the predictive power of all our biomarkers, we were able to enhance the overall predictive power of our model to 87% or better for all comparisons between clinical stages.

Unlike the other cellular respiration targets we have described, ATP5F1A autoantibodies, which bind to the α-subunit of ATP synthase responsible for ATP synthesis, were more modest predictors of clinical severity and conduction abnormalities.^48^ Like other targets we describe here, alterations in the ATP5F1A protein have been reported in a range of cardiovascular diseases including congestive HF, ischemic cardiomyopathy, and dilated cardiomyopathy, and overexpression in mice was shown to improve mitochondrial morphology and function.^49^^,^^50^ Moreover, reduced ATP5F1A expression was observed only in patients with ChCM but not in patients with idiopathic dilated cardiomyopathy or ischemic cardiomyopathy relative to controls.^51^ This reinforces the notion that ATP5F1A and its associated factors may be uniquely specific to ChCM. Even so, there are no prior reports of serum reactivity against human ATP5F1A in ChD; thus, the precise mechanism of anti-ATP5F1A formation is difficult to explain. Notably, the α-subunit of *T. cruzi* ATP synthase, which was detected in the sera of chronic Chagas patients and proposed as a diagnostic biomarker, shares ∼52% sequence homology to human ATP5F1A.^52^ This is similar to the homology between *T. cruzi enolase* and human ENO3 we outlined earlier. Combined with the fact that human ATP synthase subunits can be released in mitochondrial extracellular vesicles and potentially translocated to the plasma membrane, both of which are consistent with antigen exposure pathways, we suggest that molecular mimicry may be a plausible explanation for the observed ATP5F1A reactivity^.^^53^^,^^54^ Confirming this hypothesis with direct cross-reactivity experiments represents an important next step.

HSPA8 is involved in the removal of damaged myofibrillar proteins through the BAG3–HSPB8–HSPA8 complex at the Z-disc. Dysregulation of this axis has been associated with HF and hypertrophic cardiomyopathy.^55^^,^^56^ Immunoreactivity against HSP70 was observed in the serum of 12 of 87 patients with dilated cardiomyopathy, although it is unclear whether this detection is directly related to the cardiomyopathy or to the presence of infectious agents whose proteins elicit cross-reactive responses.^57^ In a previous investigation, no immunoreactivity against human HSP70 was detected, despite 73% similarity with the homologous protein of *T. cruzi*. Conversely, in a 2D-Western Blot analysis of human cardiomyocytes not subjected to oxidation, HSPA8 was recognized by sera from the symptomatic cardiac group, while control sera showed no reactivity.^29^ Consistently, in our cohort, individuals seropositive for ChD showed greater reactivity against recombinant human HSPA8 than the healthy group. In addition, it was possible to discriminate cardiac clinical stages within the Chagas group, with acceptable performance for distinguishing A vs C/D and normal ECG vs conduction blocks.

In our cohort, treating conduction blocks as a single phenotype of cardiac conduction system (CCS) involvement showed consistent discriminative performance and associations, which is biologically plausible in light of ChCM, in which fibrosis and inflammation compromise the AV node and the His-Purkinje system, favoring conduction disturbances.^58^ Moreover, key proteins in the CCS are not exclusive to a single segment. Connexin 40 persists in the atrial myocardium and the His–Purkinje system, and HCN4 is present from the sinoatrial node/AVN to the Purkinje fibers, supporting the notion that immune-mediated injury can affect multiple levels of the CCS even when the block manifests electrocardiographically at a specific site.^59^^,^^60^ In parallel, there is evidence of functional autoantibodies in patients with ChD, such as anti-M2, which inhibit receptor activation and are associated with autonomic dysfunction and conduction disturbances, and anti-β1-adrenergic antibodies with arrhythmogenic activity and electrophysiological modulation, providing an additional mechanism for conduction dysfunction beyond structural injury.^61^^,^^62^ Along the same lines, gap-junction remodeling in ChCM, with reduction/disorganization of Connexin 43, can slow impulse propagation and facilitate blocks.^24^ Taken together, these data support the interpretation that autoimmunity, compounded by other factors and acting on an inflammatory-fibrotic substrate, can dysregulate key protein networks distributed throughout the CCS and thereby converge on blocks in different CCS segments. Previous work on autoantibodies and conduction disorders has been inconsistent: 1 study linked high OD against the M2-muscarinic acetylcholine receptor to distal conduction disturbances (*P* = 0.03), whereas others found no significant association.^28^^,^^63^^,^^64^ These observations suggest that specific autoantibody-based analyses may help identify biomarkers for conduction abnormalities such as RBBB, LBBB, and AVB.

In summary, our results identify 6 autoantibody biomarkers (ENO1, PKM, ACTN2, MYH7, ATP5F1A, and HSPA8) that are capable of differentiating between different clinical stages of ChCM and distinct cardiac etiologies. Beyond confirming previously reported targets, our study expands the existing literature by evaluating biomarker performance against disease-free individuals and well-characterized cohorts of patients with non-Chagasic cardiac conditions, allowing a more rigorous assessment of the specificity of these biomarkers to ChCM. Notably, ENO1, ACTN2, HSPA8, and ATP5F1A have not, to the best of our knowledge, been previously validated in ChCM. Most significantly, our work is the first to demonstrate that a combined profile of autoantibodies can be linked to and provide a strong discriminatory performance for the development and worsening of clinical disease, strengthening the argument for their use as biomarkers for disease stratification and clinical management.

## Conclusions

This study supports the utility of a multitarget autoantibody panel as a serologic tool for staging and monitoring ChCM. ENO1, PKM, and ACTN2 stood out as the most promising targets, exhibiting superior performance across the entire clinical spectrum evaluated. These findings suggest that an immunologic signature is associated with disease severity, providing a basis for improved risk stratification and earlier clinical intervention. Incorporating these biomarkers into diagnostic algorithms has the potential to enhance the management of chronic ChD and guide future research into autoimmune mechanisms in ChCM.

### Study limitations and future perspectives

This study, limited by a modest single-center sample and cross-sectional design, lays the groundwork for broader investigations. Recombinant proteins from different sources were used in the validation phase because they were commercially available and standardized for immunoassay applications. However, differences in antibody-recognized epitopes between native myocardial proteins and recombinant antigens cannot be excluded and warrant further investigation. Ongoing collaborations with national and international centers aim to expand the diversity of samples, and analysis of a longitudinal cohort with over 10 years of follow-up will enable the study of temporal patterns and clinical trajectories. Although we did not explore the functional roles of the identified autoantibodies, their discovery opens the door to mechanistic studies to confirm direct binding of antibodies to cardiac tissue and to delineate molecular mimicry, autoantigen release, and cryptic epitope exposure, or both, as the source of the autoreactive antibodies. As a next step, we will evaluate an independent cohort of benznidazole-treated patients to determine whether these autoantibodies can serve as biomarkers of treatment response.

Perspectives **COMPETENCY IN MEDICAL KNOWLEDGE:** Chagas cardiomyopathy lacks reliable biomarkers for early diagnosis and risk stratification. This study identifies a panel of cardiac autoantibodies that are associated with disease severity and conduction abnormalities, and demonstrate a high diagnostic performance. We also provide evidence supporting ELISA-based serological testing as a potentially accessible strategy to identify patients at high risk and complement current clinical approaches.

**TRANSLATIONAL OUTLOOK:** Despite promising findings, immune responses may vary across populations, requiring validation in larger and more diverse clinical cohorts. Standardization of assays and establishment of clinically relevant thresholds are essential for implementation. Furthermore, a deeper understanding of the role of these autoantibodies in the pathophysiology of Chagas cardiomyopathy may enable the development of novel diagnostic and therapeutic strategies.

## Funding Support and Author Disclosures

This study was primarily supported by the 2023 TMRC Opportunity Fund, administered by RTI International with NIAID involvement (Project No. 7-312-0218586-67779L; Dr Silbiger, supervised by Dr Hamilton). Sample collection, personnel support, and related research activities at participating institutions were supported through grants and scholarships awarded to the collaborating investigators, including CAPES (Finance Code 001; Dr Silbiger, Dr Neta); CNPq (grants 442963/2023-8, HGS; 408409/2023-1, Dr Luchessi; 310790/2021-2 and 408659/2024-6, Dr Ribeiro; 310265/2021-5, Dr Dutra; and 305733/2022-2, Dr Gomes); FAPEMIG (grants RED 00192-23 and APQ-08684-25, Dr Ribeiro; APQ-04477-23 and APD-00044-25, Dr Gomes); FAPESP (grants 2022/00758-8 and 2014/50890-5, Dr Cunha-Neto); INCT-III (grant 08685/2024-7, Dr Cunha-Neto and Dr Silbiger); INCT-DT (2024, Dr Dutra); the CIHR–IDRC–ISF Joint Canada–Israel Health Research Program Phase II (IDRC grant 109929, Dr Cestari); and the University of Toronto McLaughlin Centre CREMS Scholarship (2025-2026, Dmitriev). All other authors have reported that they have no relationships relevant to the contents of this paper to disclose.
