# Supplementary material for: Identifying Cardiac Autoantibody Biomarkers that Predict the Severity of Chagas Cardiomyopathy
Source: JACC Basic Transl Sci. 2026 Oct 9;11(11):101709. doi: 10.1016/j.jacbts.2026.101709 (PMC13677190; doi:10.1016/j.jacbts.2026.101709)
Supplement: Supplementary file 1 [file mmc1.pdf]

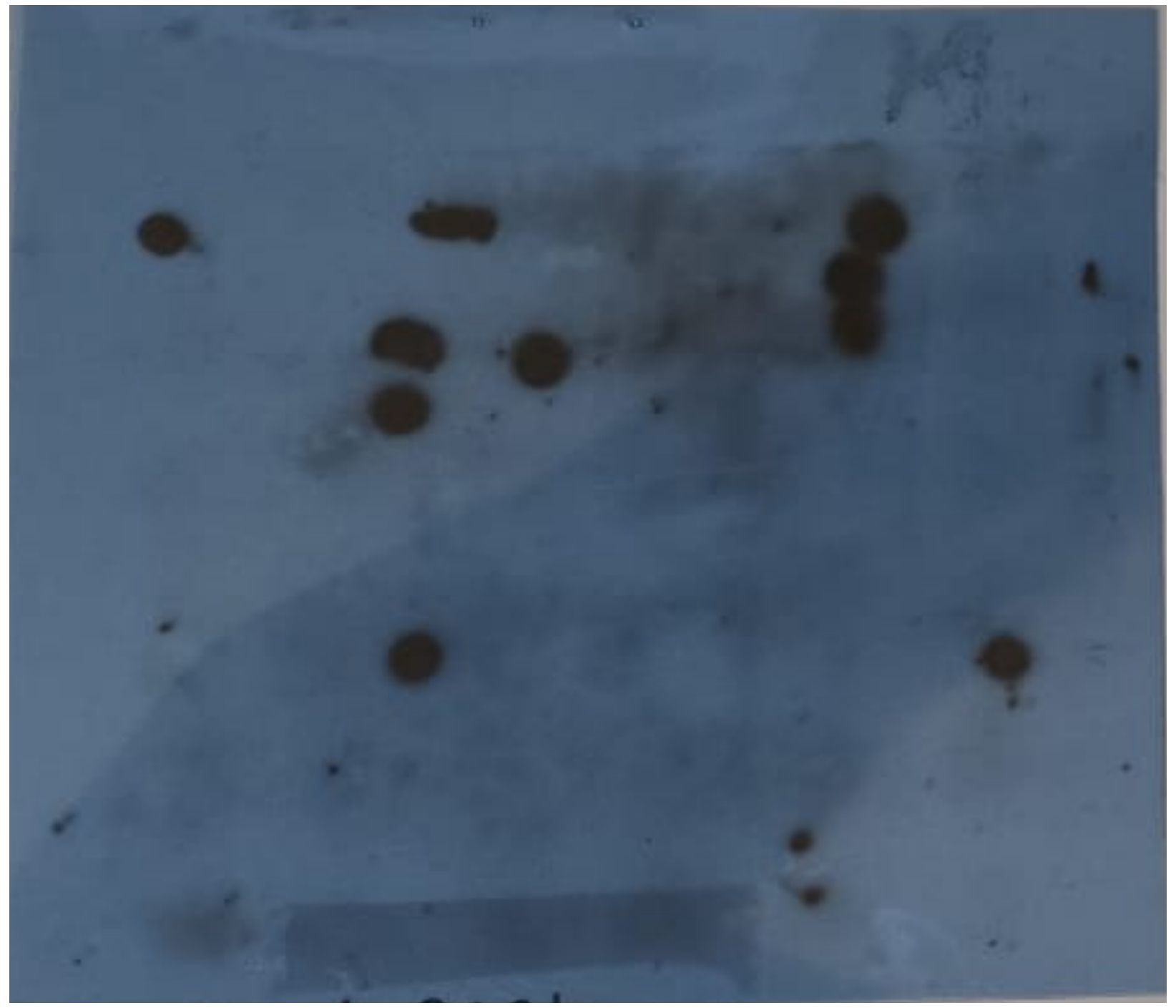

Complete unedited gel for Figure 1A

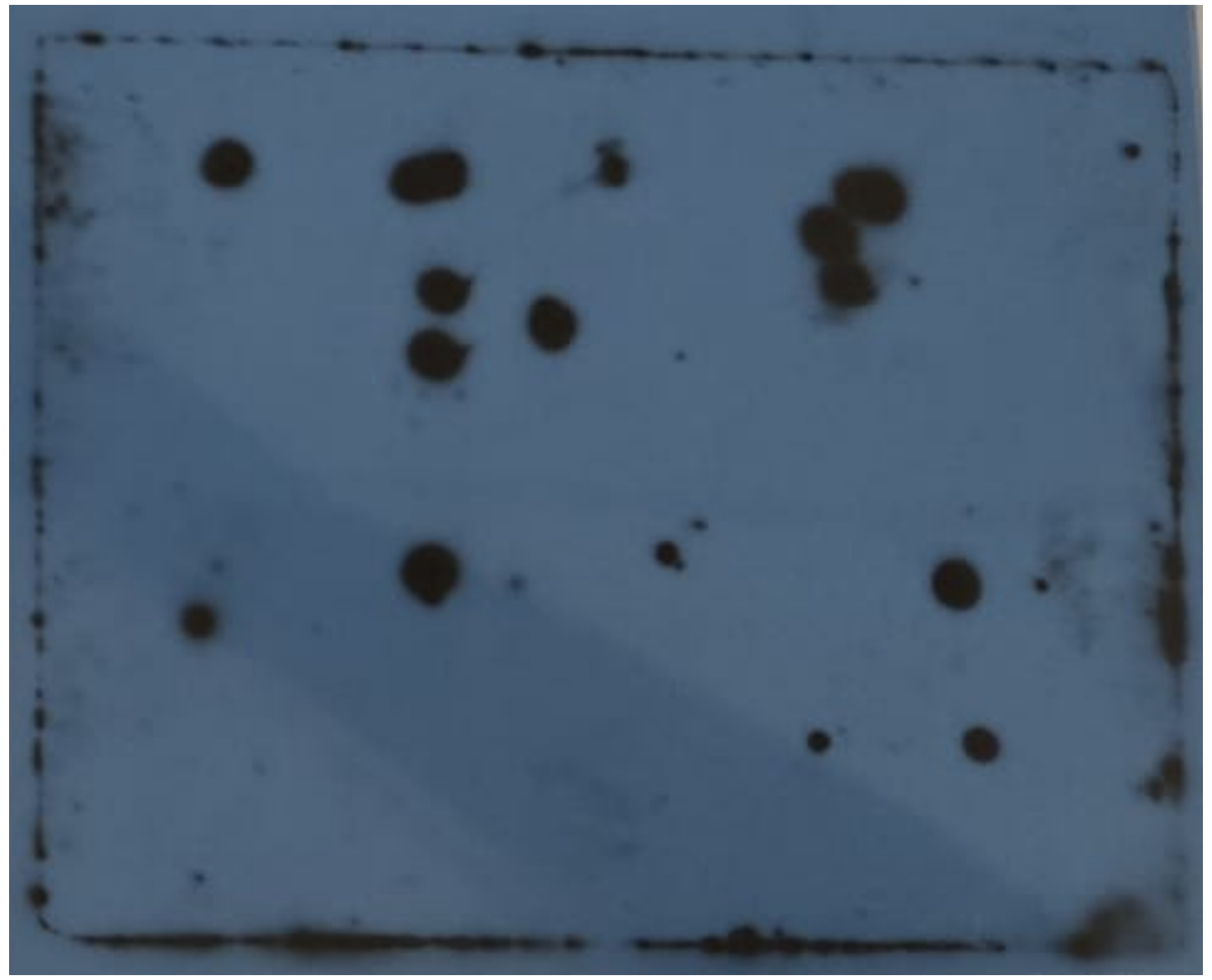

Complete unedited gel for Figure 1B

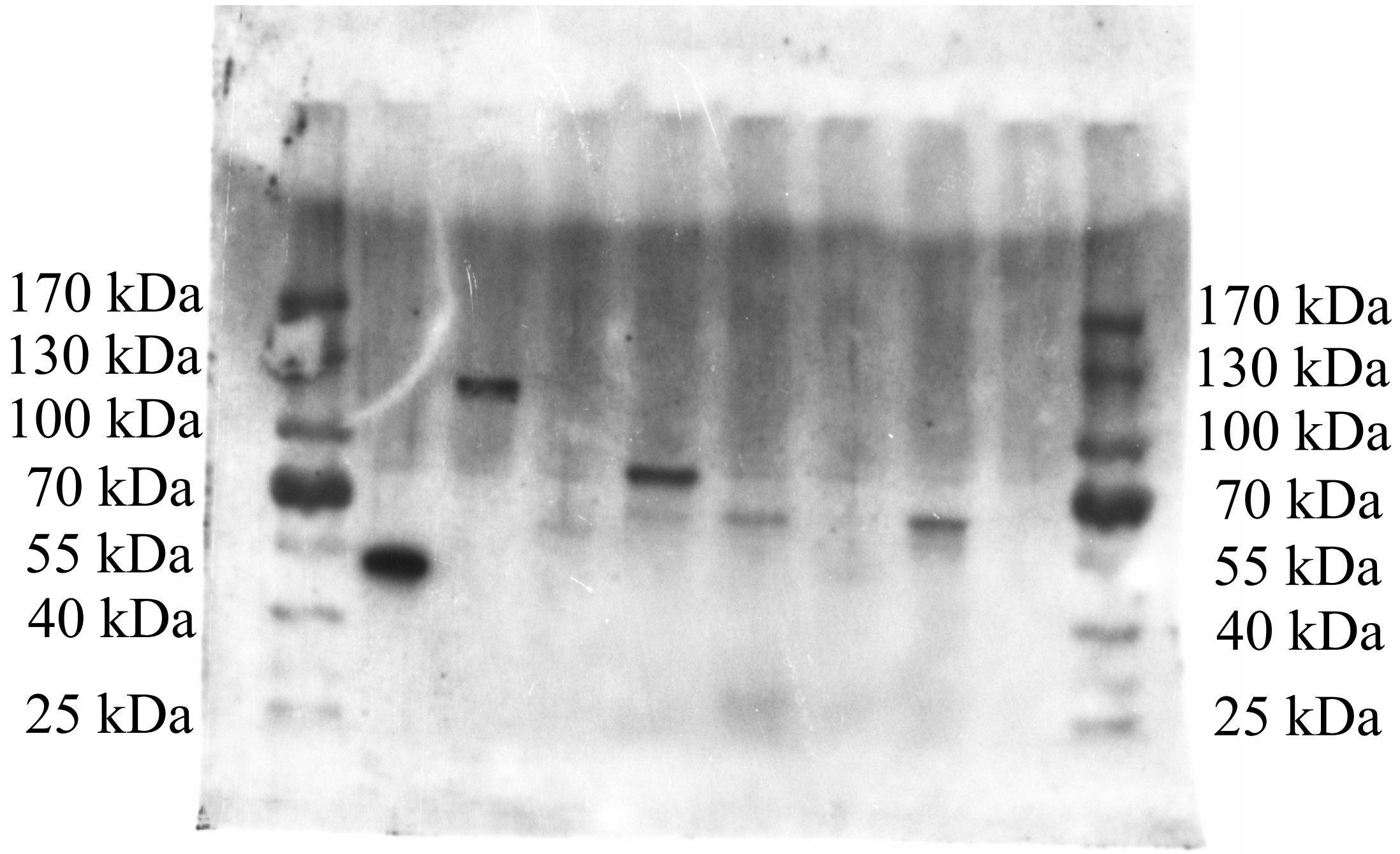

Complete unedited gel for Figure 1E

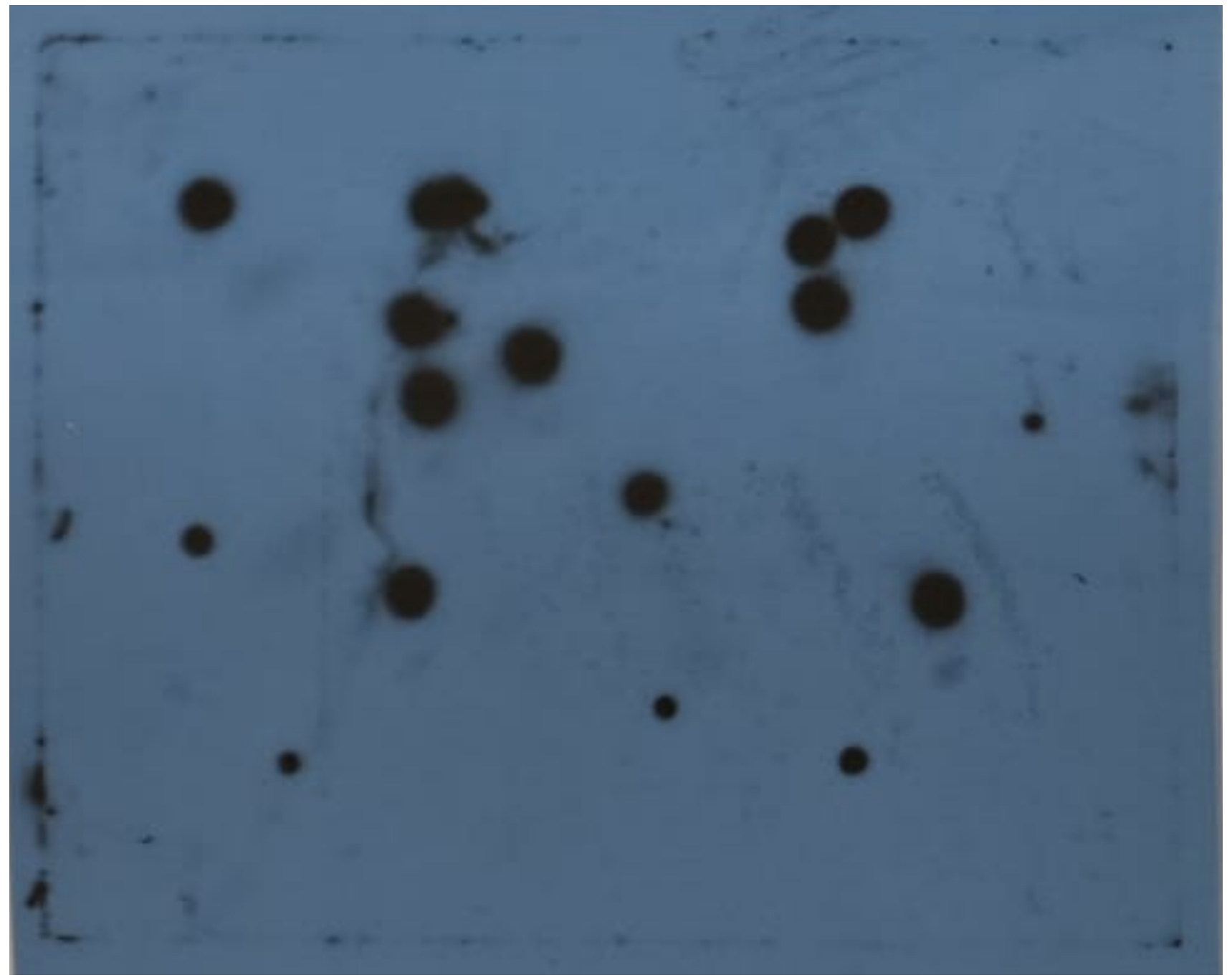

Complete unedited gel for Figure 1C

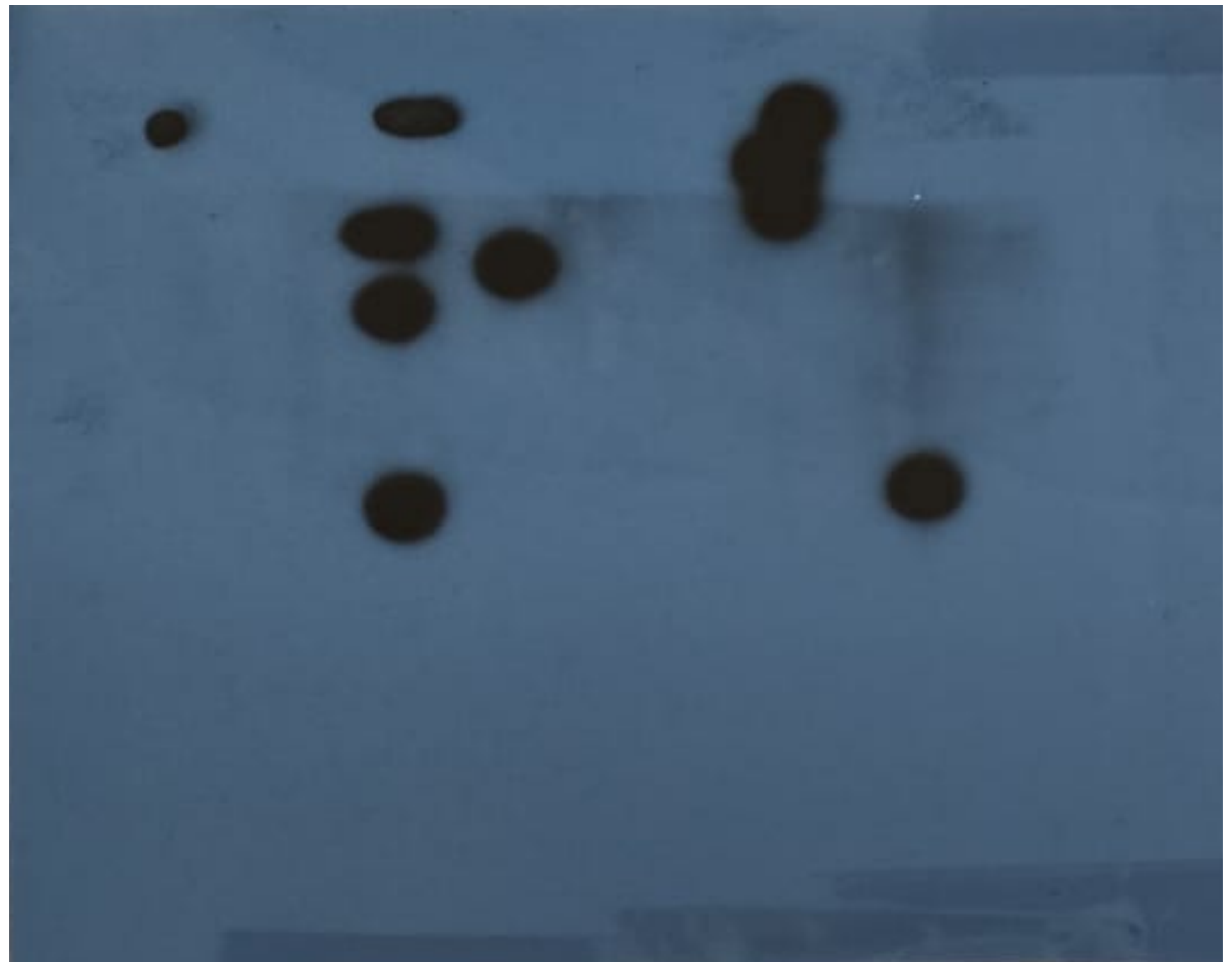

Complete unedited gel for Figure 1D
