# Supplementary material for: Identifying Cardiac Autoantibody Biomarkers that Predict the Severity of Chagas Cardiomyopathy
Source: JACC Basic Transl Sci. 2026 Oct 9;11(11):101709. doi: 10.1016/j.jacbts.2026.101709 (PMC13677190; doi:10.1016/j.jacbts.2026.101709)
Supplement: Supplementary file 2 [file mmc2.docx]

TITLE: Supplementary Figure 1**.** Autoantibodies Levels in Chagas Disease and Cardiac Disorders

LEGEND: **Supplementary Figure 1. A–F.** Comparison of serum levels (log OD) against markers across indeterminate (A), moderate (B1/B2), severe (C/D), Arrhythmogenic Right Ventricular Cardiomyopathy (ARVC), Brugada Syndrome (BrS), and Ischemic Heart Failure (HF). Statistical analysis used the Kruskal–Wallis test with post-hoc pairwise comparisons via the Benjamini, Krieger, and Yekutieli method. Adjusted p-values and sample sizes (N) are shown. *P < 0.05; **P < 0.01; ***P < 0.001.

**Supplementary Figure 1. A–F.**

**Supplementary Table 1.** Youden Cut‑offs and Odds Ratios of Candidate Autoantibody Markers for Chagas Disease Stages and Conduction Blocks

|  | **Stage A vs B1/B2** | | | | **Stage A vs C/D** | | | | **Normal ECG vs Conduction Blocks** | | | |
| --- | --- | --- | --- | --- | --- | --- | --- | --- | --- | --- | --- | --- |
| **Marker** | **Youden Cut-off** | **OR** | **95% CI** | ***p*** | **Youden Cut-off** | **OR** | **95% CI** | ***p*** | **Youden Cut-off** | **OR** | **95% CI** | ***p*** |
| ACTN2-1023H | 0.247 | 1.69 | 0.96-2.98 | 0.067 | 0.246 | 3.14 | 1.38-7.15 | **0.006** | 0.246 | 2 | 1.13-3.53 | **0.017** |
| ATP5F1A-2238H | 0.315 | 1.5 | 0.95-2.36 | 0.079 | 0.19 | 2.06 | 1.19-3.57 | **0.009** | 0.291 | 1.69 | 1.09-2.61 | **0.019** |
| ENO1-1814H | 0.209 | 1.88 | 1.14-3.11 | **0.013** | 0.205 | 1.92 | 1.18-3.12 | **0.008** | 0.459 | 1.96 | 1.22-3.17 | **0.005** |
| HSPA8-4712H | 0.279 | 1.3 | 0.98-1.72 | 0.067 | 0.205 | 1.36 | 0.98-1.87 | 0.062 | 0.25 | 1.4 | 1.04-1.9 | **0.027** |
| MYH7-8021H | 0.406 | 1.35 | 0.95-1.91 | 0.094 | 0.228 | 1.6 | 1.09-2.35 | **0.015** | 0.228 | 1.44 | 1.04-2.01 | **0.028** |
| PKM-009H | 0.33 | 1.51 | 0.97-2.35 | 0.070 | 0.232 | 1.86 | 1.15-3 | **0.010** | 0.264 | 1.68 | 1.1-2.55 | **0.015** |

Odds Ratio, OR; CI, Confidence Interval. OR and 95% CI are shown per 0.1 OD unit increment. Significant values (*p* < 0.05) are shown in bold.

**Supplementary Table 2.** Clinical information about the Arrhythmogenic Right Ventricular Cardiomyopathy cohort.

| **Clinical Variable** | **ARVC (10)** |
| --- | --- |
| Age, y, mean ± SD | 50.7 ± 11.4 |
| Sex, male n (%) | 7 (70) |
| **Task Force Criteria,** mean ± SD | 5.7 ± 1.3 |
| **Risk score parameters** |  |
| RVEF, mean ± SD | 37.2 ± 12.7 |
| PVC count, mean ± SD | 2370.9 ± 1868.8 |
| Cardiac Syncope (<6mo), n (%) | 0 (0) |
| No. of inv. T-W, mean ± SD | 4.8 ± 2.8 |
| Non-sustain VT, n (%) | 6 (60) |
| susVA (np)5, mean ± SD | 9.6 ± 7.6 |

Arrhythmogenic Right Ventricular Cardiomyopathy (ARVC), based on Task Force Criteria. RVEF, Right Ventricular Ejection Fraction; LVEF, Left Ventricular Ejection Fraction; PVC, Premature Ventricular Contraction; susVA, No. of inv. T-w: number of inverted T-wave complexes observed on an electrocardiogram; susVA (np)5: risk calculator estimating the five-year risk of sustained ventricular arrhythmias (VA) in patients with a definite ARVC diagnosis (www.arvcrisk.com);

**Supplementary Table 3.** Clinical information about the Brugada Syndrome cohort.

| **Clinical Variable** | **BrS (10)** |
| --- | --- |
| Age, y, mean ± SD | 45 ± 11.9 |
| Sex, male n (%) | 10 (100) |
| **Shanghai, score, mean ± SD** | 5.4 ± 2.2 |
| Syncope, positive n (%) | 2 (20%) |

Brugada Syndrome (BrS), based on a Shanghai Score >3.5.

**Supplementary Table 4.** Clinical information about the patients with previous myocardial infarction and left ventricular systolic dysfunction (LVEF ≤45%), consistent with heart failure (HF).

| **Clinical Variable** | **Post-AMI patients with LV systolic dysfunction (n = 21)** |
| --- | --- |
| Age, years | 56.7 ± 11.8 |
| Male sex | 18 (85.7) |
| Body mass index, kg/m² | 29.4 ± 5.6 |
| Obesity (BMI ≥30 kg/m²) | 8 (38.1) |
| Hypertension | 18 (85.7) |
| Diabetes mellitus | 10 (47.6) |
| Dyslipidemia | 10 (47.6) |
| Current/former smoking | 11 (52.4) |
| Current/former alcohol consumption | 8 (38.1) |
| Regular physical activity | 13 (61.9) |
| Systolic blood pressure, mmHg† | 109.2 ± 14.1 |
| Diastolic blood pressure, mmHg† | 69.8 ± 8.3 |
| Left ventricular ejection fraction, % | 38.0 ± 5.0 |
| HFrEF (LVEF <40%) | 11 (52.4) |
| HFmrEF (LVEF 40–49%) | 10 (47.6) |
| HFpEF (LVEF ≥50%) | 0 (0.0) |
| **NYHA functional class** |  |
| I | 7 (33.3) |
| II | 1 (4.8) |
| III | 4 (19.0) |
| IV | 1 (4.8) |
| Not documented | 8 (38.1) |

Values are presented as mean ± SD or n (%). All participants had documented left ventricular systolic dysfunction (LVEF ≤45%) following acute myocardial infarction (AMI). Smoking was defined as current or former smoking, and alcohol consumption included both current and former alcohol use. Regular physical activity was defined based on self-reported engagement in routine exercise. NYHA functional class was extracted from the patients' medical records; eight patients had no documented NYHA classification. Blood pressure measurements were available for 17 of 21 patients. When multiple blood pressure measurements were documented, the highest recorded systolic blood pressure and its corresponding diastolic blood pressure were used for analysis. HFmrEF = heart failure with mildly reduced ejection fraction; HFpEF = heart failure with preserved ejection fraction; HFrEF = heart failure with reduced ejection fraction; LVEF = left ventricular ejection fraction; NYHA = New York Heart Association.
